# Nutrient digestibility, organ morphometry and performance in vaccinated or non-vaccinated *Lawsonia intracellularis* infected piglets

**DOI:** 10.1186/s12917-018-1662-2

**Published:** 2018-11-01

**Authors:** Christian Visscher, Jasmin Mischok, Saara Sander, Marion Schmicke, Eva-Ursula Peitzmeier, Isabel von dem Busche, Karl Rohn, Josef Kamphues

**Affiliations:** 1 Institute for Animal Nutrition, University of Veterinary Medicine Hannover, Foundation, Bischofsholer Damm 15, D-30173 Hannover, Germany; 2 Clinic for Cattle, University of Veterinary Medicine Hannover, Foundation, Bischofsholer Damm 15, D-30173 Hannover, Germany; 3 Tierarztpraxis Dr. Peitzmeier, Meente 24, D-32479 Hille, Germany; 4 Institute for Biometry, Epidemiology and Information Processing, University of Veterinary Medicine Hannover, Foundation, Bünteweg 2, D-30559 Hannover, Germany

**Keywords:** *Lawsonia intracellularis* infection, Vaccination, Digestibility, Performance, IGF-1, Organ morphometrics

## Abstract

**Background:**

*Lawsonia intracellularis* is one of the world’s most important infectious diseases in pork production with regard to economic losses. So far, studies are missing that describe the effects of a natural infection of piglets on the digestibility of nutrients, possible effects on performance and the morphometrics of the intestine depending on whether piglets are vaccinated, clinically healthy or clinically affected with regard to *Lawsonia intracellularis* induced diarrhoea.

**Results:**

Digestibility studies were performed on a total of 27 eight-week-old piglets with naturally occurring *Lawsonia intracellularis* infection in a trial with three repetitions. Nine out of 27 animals were vaccinated as suckling pigs with a commercial *Lawsonia intracellularis* vaccine (vac; Enterisol®Ileitis). Half of the remaining 18 animals were without clinical signs of infection (non-vac/cs-), half showed moderate clinical signs of *Lawsonia intracellularis* induced diarrhoea (non-vac/cs+). All three groups were fed one identical complete diet *ad libitum*. Faecal shedding of *Lawsonia intracellularis* was found in all groups (25 out of 27 animals). Numerically, the mean excretion in the group non-vac/cs + (7.69 ± 1.65 log_10_ copies/ g faeces) was higher in comparison to the group non-vac/cs- (5.83 ± 2.35 log_10_ copies/ g faeces) and vaccinated animals (vac: 6.00 ± 2.89log_10_ copies/ g faeces). The average daily weight gain (ADG; Ø 8.66 day period) differed significantly (vac: 894^a^ ± 73.3, non-vac/cs-: 857^ab^ ± 86.3, non-vac/cs+: 785^b^ ± 137 g/day). The apparent total tract digestibility (ATTD) of nitrogen was significantly lower in clinically affected animals (vac: 83.0^a^ ± 1.72, non-vac/cs-: 83.9^a^ ± 2.03, non-vac/cs+: 80.7^b^ ± 2.57).The total length of the small intestine in clinically affected animals increased significantly (vac: 15.9^ab^ ± 1.57, non-vac/cs-: 14.6^b^ ± 1.12, non-vac/cs+: 16.2^a^ ± 1.37 m). The relative body weight depending on the length of the small intestine was lower for clinically affected animals (vac: 1.72^a^ ± 0.21, non-vac/cs-: 1.83^a^ ± 0.17, non-vac/cs+: 1.56^b^ ± 0.12 kg/m).

**Conclusion:**

These studies show that clinically moderate *L. intracellularis* infections lead to significantly lower ADGs in comparison to vaccinated animals. The disease is also found in altered intestinal morphometry and reduced total N digestibility if clinical signs occur.

## Background

Infections with *Lawsonia intracellularis* (*L. intracellularis*) in pigs lead to economic losses both in the case of clinical manifestations and subclinical infections [1–4]. Lower performance results from reduced growth and an impaired feed efficacy [5–8]. The overall prolonged rearing and fattening period is a typical finding of subclinical infections [1, 9–11]. This is a common sign of an *L. intracellularis* infection in practice [2]. Clinically apparent infections are traditionally associated with diarrhoea and can be related to animal losses due to the haemorrhagic course named porcine haemorrhagic enteropathy [4, 12]. These may occur more frequently in individual stocks, but play a less important role overall [13].

In order to convert the absorbed nutrients at their highest rate into weight gain, a good state of health of the growing animals is essential. This excludes any avoidable activation of an inflammatory immune response, which might also be common for gastrointestinal infections [14]. High concentrations of proinflammatory cytokines often lead to a condition known as the systemic inflammatory response syndrome (SIRS) [15] which is often related to sickness symptoms like decreased feed and water intake [15, 16]. The reduced appetite is mediated by these aforementioned cytokines acting in the central nervous system [15–17]. There are findings indicating that Insulin-like growth factor 1 (IGF-1) is potent in attenuating sickness behaviour induced by tumour necrosis factor-alpha [17]. On the other hand there are indications that the immune response is hardly activated in the initial course of an infection with *L. intracellularis* [18].

In general, an immediate reduction in average daily feed intake (ADFI) for pigs affected by digestive bacterial infections can be estimated at 15% [19]. Nonetheless, also an impaired digestibility of the nutrients has to be considered in cases of an *L. intracellularis* infection. With regard to the pathomorphological changes in the intestines of infected pigs, it has to be assumed that due to the reduced permeability of the intestinal wall there is a reduced absorption of certain nutrients in cases of an *L. intracellularis* infection [20, 21]. For infections with porcine epidemic diarrhoea virus apparent total tract digestibility (ATTD) of dry matter (DM) and energy are decreased by 8 and 12%, respectively [22]. However, the digestibility of nutrients and the concentrations of nutrients in the digesta of the terminal ileum influence the length of the gastrointestinal tract [23]. This is particularly noticeable after weaning because here very fast adaptation can be seen in the form of elongation [23]. In general, the response of the gastrointestinal tract to ingestion of nutrients is a complex and closely controlled process [24]. Mechanical and chemical control mechanisms are involved in the short and long term. Due to the feed consumption there is a distension of hollow viscera along with activation of both elongation and tension mechanoreceptors [24]. There are simultaneously nutrient-sensing chemoreceptors in the upper gastrointestinal tract [24]. These are involved in detecting the presence of all main categories of nutrients in the lumen [24]. A leading mechanism of intestinal adaption to enteral nutrients is driven by humural factors like the intestinotrophic peptide glucagon-like peptide-2 (GLP-2) [25, 26]. Enteroendocrine L-cells secrete GLP-2 after direct stimulation by nutrients distally in the small intestine [25].

To date, no investigation has been carried out on how an infection with *L. intracellularis* might affect the digestibility of nutrients, performance, IGF-1 status and gastrointestinal tract morphometry as a result of natural exposure when non-vaccinated and vaccinated animals are compared.

## Methods

Animal experiments were carried out according to German regulations. It was not an animal experiment requiring a notification or an approval according to the Animal Protection Act (§ 7, paragraph 2, sentence 3). Interventions before dissection were not carried out. The animals were killed according to § 4, paragraph 3 of the Animal Protection Act, exclusively to use their organs or tissues for scientific purposes.

### Origin of animals, preparation and selection

All animals used in these trials came from one farm with 420 sows of Danish genetics (DK: Danish Landrace 50% x Yorkshire 50%). In total, 27 piglets (DK x Pietrain) were used in a trial with three repetitions. The sows in the herd were regularly vaccinated (Porcine Reproductive and Respiratory Syndrome Virus, Swine Influenza Virus, Porcine Parvovirus, *Erysipelothrix rhusiopathiae*, *Escherichia coli* + *Clostridium perfringens* dam vaccine) as were the piglets (Porcine Circovirus 2, *Mycoplasma hyopneumoniae* and *Haemophilus parasuis*). In the context of regular and long-term screening programmes, the herd showed no signs of *Salmonella* and *Brachyspira* infections. Clinical symptoms of an *L. intracellularis* infection (confirmed by pathogen detection in faeces) were, on the other hand, a common finding in piglets at an age of seven to nine weeks. Before the starting the experimental trials, additional results of serological analyses of piglets were obtained from the veterinarian of the herd. From these random tests in the rearing phase of piglets, it was found that there were no serological positive animals in the middle of this phase whereas at the end of the rearing phase, only 10% were still serological negative (data not shown).

The production system on the farm was organised in a two-week rhythm. During the trial period every two weeks approximately 50 piglets from a total of four to five complete litters were vaccinated with a commercial *L. intracellularis* vaccine (Enterisol^®^Ileitis, Boehringer Ingelheim Vetmedica GmbH, Ingelheim / Rhine, Germany) on the 21st day of life. The vaccination was done via oral drenching. Subsequently, the piglets were marked individually.

**Fig. 1:**
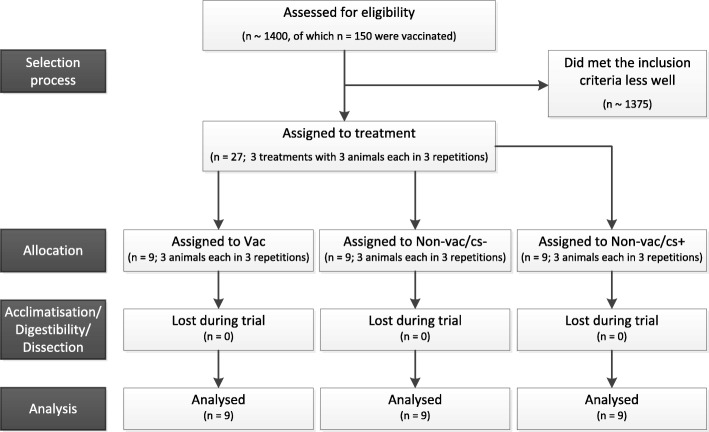
Clinical trial flow diagram. The diagram indicates selection process and loss of animals during the trial

At weaning all 450 to 500 piglets in a group were placed in a piglet rearing compartment in groups of 12 to about 30 animals on fully slatted floors (Fig. 1). Non-vaccinated and vaccinated animals were mixed. From weaning to the presumed initial *L. intracellularis* infection time point, samples from the individual stables were tested for their *L. intracellularis* status by real-time PCR using established methods [27]. With the positive proof a change to a single-animal examination procedure took place. All piglets in a weaning group were clinically examined. Individual samples were collected from conspicuous animals with regard to faecal quality (healthy pigs with moderate to soft feaces consistency) and analysed by means of real-time PCR [27]. After obtaining the results, three conspicuous animals (no siblings) were taken from the appropriate weaning group (non-vac/cs+). At the same time three clinically healthy ones (non-vac/cs-) and three vaccinated animals (vac+) of the same age and identical weight were chosen from the group, if possible in a balanced gender relationship. All selected animals were transported to the Institute for Animal Nutrition, University of Veterinary Medicine Hannover, Foundation, Hannover, Germany.

### Animal husbandry and experimental conditions

The pigs were housed individually in 3 × 1 m boxes during the entire trial period. The boxes were equipped with a nipple drinker, a 1 m long earthenware trough at one of the narrow sides of the box, an infrared warming lamp and (manipulable material). Visual contact to other animals was possible at all times. The boxes had a concrete floor. All three repetitions of the trial were performed identically.

### Diet and digestibility trials

The pigs continued to receive the diet they were accustomed to on the farm. The diet contained 41.0% wheat, 35.0% barley, 18.0% soybean meal, 2.00% soybean oil and 4.00% of a mineral and vitamin supplement (MOVIKALIN Speed 10^®^, HS-Kraftfutterwerk, Ochtrup, Germany; Table 1).

**Table 1:** Energy content and concentrations of ingredients in the diet for rearing piglets

Item		Content
Metabolisable Energy (ME, MJ)	per kg diet	13.8
Crude ash	g/kg DM	56.7
Crude fat		38.4
Crude fibre		26.9
Crude protein		201
Starch		480
Sugar		21.6
Lysin		16.5
Ca		9.88
P		6.38
Na		2.14
Cu	mg/kg DM	156
Zn		150
Se		0.44

Mineral and Vitamin supplement (4% of complete diet) contained 500,000 I.E Vit A, 50000 I.E. Vit. D3, 3000 mg Vit. E, 30 mg Jodine

The nutrient composition was in accordance with the official recommendation for piglets in Germany. The complete diet for all trial repetitions derived from one batch. The digestibility trial consisted of two phases starting with a 3-day adaptation period followed by a five-day collection period. The adaptation period was shortened to only three days due to the use of the identical diet already on the farm. In this way, the experiment still focused on the clinically conspicuous phase of the natural *L. intracellularis* infection. Chromium oxide (0.5%) was added to the diet as a marker for calculating the precaecal digestibility. The diet was offered *ad libitum*. The feed was changed once a day. Feed refusals were removed and the amount determined after drying at 103 °C. The collected faeces were weighed by means of electronic scales (Acculab, Sartorius AG, Göttingen, Germany) and then frozen. At the end of the collection period, an aliquot of the total amount of faeces was collected after thawing and homogenisation for further analysis.

### Dissection

At day of the dissection the pigs were fed in a timely manner. There was an interval of nine hours between offering the fresh diet and death of each animal. In each repetition 3 out of 9 animals were dissected on the day of finishing the digestibility trial, the remaining animals on the following day. Prior to the dissection the animals were anaesthetised with neuroleptanalgesia by means of a combination of ketamine (Ursotamin^®^ 10%, Serumwerke Bernburg, Bernburg, Germany; active ingredient: ketamine hydrochloride, dosage: 15 mg/kg body weight intramuscularly) and azaperone (Stresnil^®^4% Janssen Animal Health, Neuss, Germany; active ingredient: azaperone, dosage: 2 mg/kg body weight intramuscularly). In the course of the dissection a blood sample was taken intracardially from the pigs. After that, pigs were euthanised with T61®intracardially (Intervet, Unterschleißheim, Germany; active ingredients: tetracaine hydrochloride, embutramide, mebenzonium iodide, dosage: 0.6 mL/10 kg body weight).

Before opening the abdominal cavity the naso-rump length of the animals was measured. The abdominal cavity was opened along the linea alba as well as a relief cut being performed in the flank region. The caecum was pre-placed, double-ligated and then removed. Other ligatures were placed directly between the pylorus and the jejunum and in front of the rectum. The removal of the stomach was done by dull preparation of the diaphragm and connective tissue. After placing an intestinal clamp on the cardia and severing the connection to the jejunum, the stomach was removed. Subsequently, the removal of the whole intestinal tract and the application of a ligature between the colon and the jejunum took place. Both the colon and the jejunum were freed from the mesenterium and their total length was measured in the filled state, also the length of the blind arm was measured. Digesta from the terminal small intestine for precaecal digestibility determination was taken in accordance with common procedures [22]. The contents of the different segments (stomach, small intestine, caecum, colon) were collected separately in plastic containers and used for further analysis.

### Analytical methods

Diets were analysed by standard procedures in accordance with the official methods of the VDLUFA [28].The analyses were always performed in duplicate. The dry matter content was determined by drying to the weight constancy at 103 °C. The crude ash was analysed by means of incineration in the muffle furnace at 600 °C for 6 h. The total nitrogen content was determined by means of the analyser Vario Max^®^ (Elementar, Hanau, Germany), which operates according to the principle of a catalytic tube combustion (DUMAS combustion method). The molecular nitrogen formed by reduction from nitric oxide was detected by a thermal conductivity detector and the nitrogen content was calculated by the device software. The crude protein content of the sample was calculated by multiplication with a constant factor of 6.25. The crude fat content was determined after acid hydrolysis in the Soxhlet apparatus. The content of crude fibre was determined after washing in diluted acids and alkalis by established methods. The determination of starch contents was carried out polarimetrically (Polatronic E, Schmidt und Haensch GmbH & Co., Berlin, Germany). The sugar content was analysed according to Luff-Schoorl by titration with sodium thiosulphate. The mineral content was determined in accordance with the official methods [28] by atomic absorption spectrometry (Unicam Solaar 116, Thermo, Dreieich, Germany). The analyses of the chyme were performed in principle by the identical methods as described for feeds.

The determination of mean *L. intracellularis* genome equivalents (GE) was done from the aliquot of the homogenised faeces via quantitative PCR according to established methods [29]. The numbers are shown in the logarithm of 10.

Insulin-like growth factor I (IGF-1) concentration was determined using a commercial radioimmunoassay acoording to the standard operation manual (A15729, IRMA IGF-I; Beckman Coulter, CA, USA). The assay was validated for porcine plasma by determining the recovery and an intra-assay coefficient of variation by measuring 20 times one sample within one test routine. The recovery was 94,5% and the intra-assay was 6,3%.

### Statistical analyses

Normal distribution of the model residuals was confirmed by Kolmogorov-Smirnov-Test and visual assessment of q-q - plots. All data were included into a descriptive analysis with calculation of the arithmetic mean, standard deviation, minimum and maximum.

The influence of the factor group (vac, non-vac/cs-, non-vac/cs+) on the quantitative variables (Log10 genome equivalents; DM faeces, performance parameters, digestibility nutrients, organ morphometry parameters) was carried out by a one way variance analysis of variance (ANOVA) with REGWQ (Ryan-Einot-Gabriel-Welsch multiple Range-Test) post hoc test for multiple pairwise comparisons, concerning the experimentwise errorrate.

Analyses were carried out with the statistical software SAS, version 9.3 (SAS Institute, Cary, NC, USA), using SAS^®^ Enterprise Guide 5.1. Differences were taken to be statistically significant when *P* < 0.05.

## Results

The investigations were carried out on a total of 27 piglets. In all three repetitions of the trial, the general health status of each animal was checked at least twice a day. The 27 animals showed an undisturbed general state. Moderate clinical diarrhoea in all animals of the non-vaccinated, clinically affected group (non-vac/cs+) previously found on the farm was included in the study as a selection criteria (moderate diarrhoea induced by *L. intracellularis*).

### Lawsonia intracellularis excretion and performance

Faecal shedding of *L. intracellularis* was found in all groups (25 out of 27 animals). There were no statistically significant differences in the absolute level of pathogen excretion (Table 2). Numerically, the mean excretion in the groups non-vac/cs- and vac was lower (− 1.87 or rather − 1.70 log units/g faeces). The clinically affected animals showed at the same time significantly lower faecal dry matter compared to the other two groups (− 10.6% compared to vac and − 13.9% compared to the group non-vac/cs-). Neither at the trial’s start nor at the time of the dissection did the body masses differ between the three groups. The daily feed intake did not differ significantly between groups. Numerically, differences between the groups could be seen. The feed intake in the group non-vac/cs + was reduced by 10.2% and in group non-vac/cs- by 6.94% compared to the feed intake of vaccinated animals. However, the sample size included only 2 out of 3 trials. The average daily body weight gains (ADG) were significantly different between the clinically affected (non-vac/cs+) and the vaccinated group (vac). In the former, the growth was reduced by 12.2%. The non-vaccinated, clinically healthy animals showed no significant differences in average daily gains compared to one of the other groups. These were only 4.14% lower than for the group vac. The FCR did not differ between the groups.

**Table 2:** Overview on Lawsonia intracellularis excretion, dry matter content of faeces and growth performance

Item	Vac		Non-vac/cs-		Non-vac/cs+	
	Mean	SD	Mean	SD	Mean	SD
Log_10_ genome equivalents	6.00	2.89	5.83	2.35	7.69	1.65
DM faeces (g/kg DM)	23.6^a^	1.84	24.5^a^	1.68	21.1^b^	1.98
BW start (kg)	19.2	1.64	19.2	1.36	18.4	1.53
BW Dissection (kg)^c^	27.0	1.71	26.7	2.19	25.3	2.43
ADFI (kg DM/day)^d^	1.297	0.116	1.207	0.119	1.165	0.148
ADG (kg/day)	0.894^a^	0.073	0.857^ab^	0.086	0.785^b^	0.137
FCR (kg diet/kg ADG)	1.410	0.075	1.422	0.078	1.470	0.061

*Vac* vaccinated with Enterisol^®^Ileitis, *Non-vac/cs-* not vaccinated, clinically no signs, *Non-vac/cs +* not vaccinated, clinical signs

^a,b^Values within a row with different superscripts differ significantly at *P* < 0.05

^c^Dissection was done in 3 out of 9 animals 8 days after starting experiment, in 6 out of 9 piglets 9 days after start of experiment

^d^Due to technical detection problems, three values are missing from one of three repetitions

**Fig. 2:**
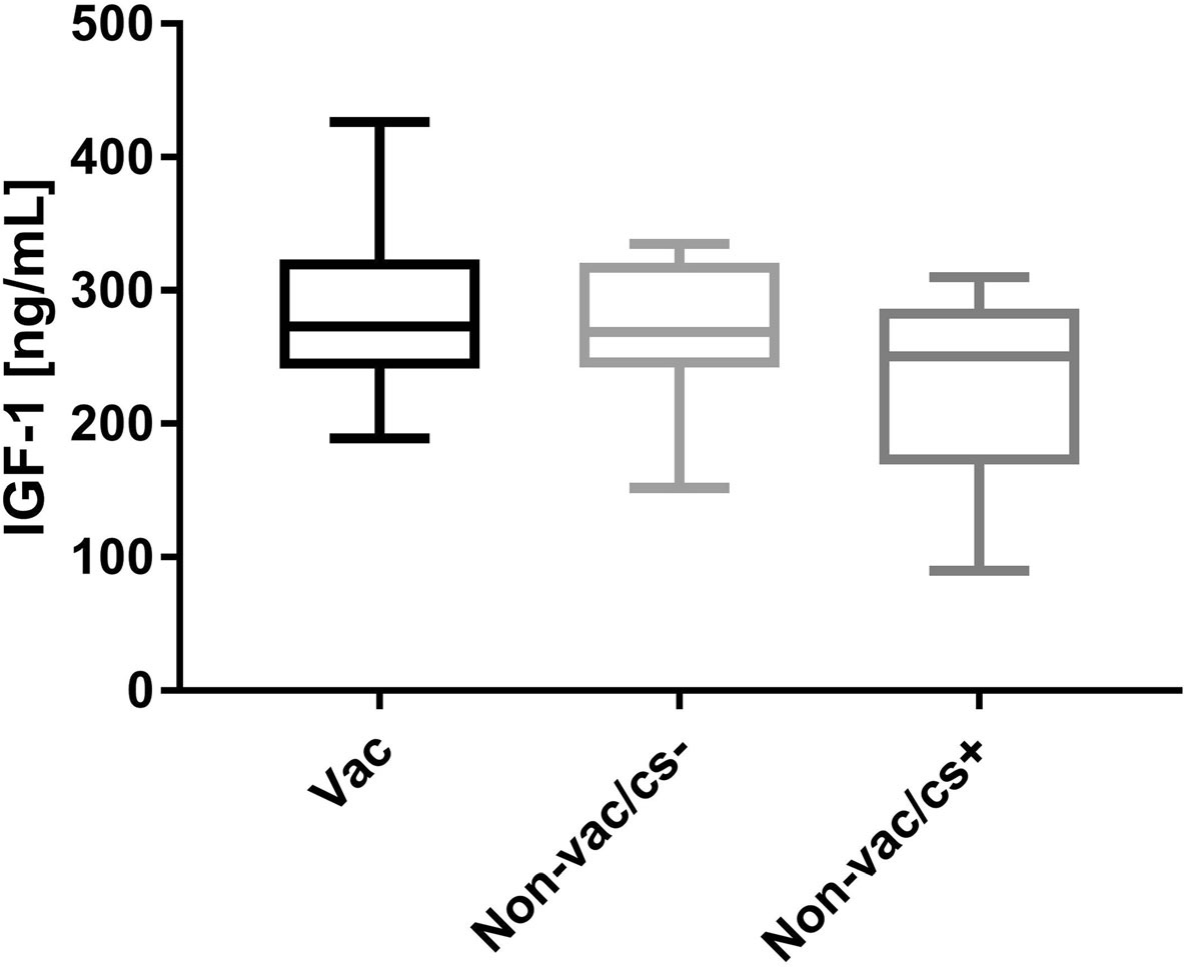
Concentrations of IGF-1 levels in blood at dissection

In the animals’ blood at the time of dissection the concentrations of IGF-1 were measured (Fig. 2). There were no significant differences in the IGF-1 levels between the three groups (Mean ± SD in ng/mL; vac: 285 ± 68.3; non-vac/cs-: 272 ± 57.1; non-vac/cs+: 226 ± 74.4).

### Digestibility of nutrients

There were no differences between the groups in regard to the precaecal digestibility of nutrients (Table 3).

**Table 3:** Apparent precaecal digestibility values of organic matter, crude protein and starch in piglets

Diet component	Apparent precaecal digestibility rates (%)					
	Vac		Non-vac/cs-		Non-vac/cs+	
	Mean	SD	Mean	SD	Mean	SD
Organic matter	62.2	7.94	66.7	2.03	64.7	5.09
Crude protein	71.6	5.18	72.5	3.93	70.2	4.25
Starch	91.3	2.32	93.2	1.97	93.3	2.31

*Vac* vaccinated with Enterisol^®^Ileitis, *Non-vac/cs-* not vaccinated, clinically no signs, *Non-vac/cs +* not vaccinated, clinical signs

Values within a row with different superscripts differ significantly at *P* < 0.05 (significances are missing)

The ATTD of the nutrients differed significantly only with respect to the crude protein (Table 4). The group with clinically affected animals showed a significantly lower N-digestibility (− 2.77% compared to group vac; − 3.81% compared to non-vac/cs-). The digestibility of organic matter, raw fat, crude fibre and starch did not differ significantly.

**Table 4:** Apparent total tract digestibility of organic matter and nutrients in piglets

Diet component	Apparent total tract digestibility (%)					
	Vac		Non-vac/cs-		Non-vac/cs+	
	Mean	SD	Mean	SD	Mean	SD
Organic matter	86.4	1.46	86.9	1.81	84.8	2.19
Crude fat	72.1	4.08	73.1	6.15	71.5	4.31
Crude fibre	18.8	7.64	22.9	10.2	14.4	15.3
Crude protein	83.0^a^	1.72	83.9^a^	2.03	80.7^b^	2.57
Starch	98.9	0.27	99.0	0.15	98.8	0.26

*Vac* vaccinated with Enterisol^®^Ileitis, *Non-vac/cs-* not vaccinated, clinically no signs, *Non-vac/cs +* not vaccinated, clinical signs

^a,b^Values within a row with different superscripts differ significantly at *P* < 0.05

### Morphometry of organs

The total length of the small intestine in clinically conspicuous animals was significantly longer (+ 1.60 m against group non-vac/cs-; Table 5). In relation to the nose-rump length, the body mass was significantly higher in vaccinated animals (+ 7.8% per m). That means, it is concentrated more mass per unit of length. Also, the clinically inconspicuous animals had a significantly higher body mass per m length of the small intestine (+ 17.3% per m) in relation to clinically conspicuous animals.

**Table 5:** Morphometric parameters and relations between morphometric parameters as well as relations to performance in piglets

	Total value and relations of parameters					
Parameter /Organ	Vac		Non-vac/cs-		Non-vac/cs+	
	Mean	SD	Mean	SD	Mean	SD
Length (m)						
Nose-rump	0.89	0.03	0.90	0.03	0.90	0.05
Small intestine	15.9^ab^	1.57	14.6^b^	1.12	16.2^a^	1.37
Caecum	0.22	0.02	0.21	0.02	0.21	0.01
Colon	3.03	0.39	2.98	0.28	2.96	0.31
Relation body weight to nose rump and organ length (kg/m)						
Nose-rump	30.4^a^	1.44	29.7^ab^	1.59	28.2^b^	2.02
Small intestine	1.72^ab^	0.21	1.83^a^	0.17	1.56^b^	0.12
Caecum	123	7.83	126	18.9	123	11.3
Colon	8.98	0.95	9.03	1.11	8.60	1.03
Relation ADG to nose rump and organ length (g/m)						
Nose-rump	1010^a^	78.6	953^ab^	71.7	875^b^	128
Small intestine	57.1^a^	9.44	58.7^a^	6.57	48.3^b^	6.85
Caecum	4070	300	4056	635	3808	622
Colon	299	45.9	289	35.1	267	48.7

*Vac* vaccinated with Enterisol® Ileitis, *Non-vac/cs-* not vaccinated, clinically no signs, *Non-vac/cs +* not vaccinated, clinical signs

^a,b^Values within a row with different superscripts differ significantly at *P* < 0.05

The vaccinated animals had the highest ADG (+ 15.4% per m) in proportion to the nose-rump length. Therefore, per unit body length more gain in body mass is concentrated. The vaccinated and also the clinically inconspicuous animals each had the highest ADG in relation to the length of the small intestine (+ 18.2% per m and + 21.5% per m).

Total mass of the caecum tissue (− 19.5% against group vac; − 17.3% against non-vac/cs-; Table 6) was significantly lower in clinically affected animals. Consequently, the relative body mass per mass of caecum was significantly increased in the presence of clinical signs of an *L. intracellularis* infection (+ 18.2%) relative to vaccinated animals. Vaccinated animals had the highest ADG (+ 13.0%) in relation to the mass of the stomach. There was a significantly reduced amount of stomach content (− 32.0% compared to group vac; − 31.4% compared to non-vac/cs-) in clinically conspicuous animals.

**Table 6:** Organ mass and relation of body weight and average daily gains to mass of organs

Parameter / Organ	Total value and relations of parameters					
	Vac		Non-vac/cs-		Non-vac/cs+	
	Mean	SD	Mean	SD	Mean	SD
Mass (g)						
Stomach	236	31.2	243	22.6	230	22.1
Small intestine	1173	82.7	1142	124	1165	173
Caecum	74.8^a^	8.94	72.8^a^	14.6	60.2^b^	9.41
Colon	418	78.9	439	57.8	400	58.0
Relation body weight to organ mass (g/g)						
Stomach	115	10.6	110	7.40	110	7.46
Small intestine	23.1	1.95	23.5	2.60	22.0	2.72
Caecum	363^b^	32.2	376^ab^	54.0	429^a^	71.6
Colon	66.3	11.6	61.3	4.65	64.2	9.48
Relation ADG to organ mass (g/g)						
Stomach	3.83^a^	0.48	3.53^ab^	0.31	3.39^b^	0.34
Small intestine	0.76	0.06	0.75	0.09	0.68	0.12
Caecum	12.0	1.00	12.0	1.67	13.3	3.15
Colon	2.22	0.50	1.96	0.17	1.99	0.43

*Vac* vaccinated with Enterisol®Ileitis, *Non-vac/cs-* not vaccinated, clinically no signs, *Non-vac/cs +* not vaccinated, clinical signs

^a,b^Values within a row with different superscripts differ significantly at *P* < 0.05

There were no significant differences between the groups regarding the ratio of the mass of the small intestine and the length of the small intestine. Numerically, the relation was highest for the non-vaccinated, clinically non-affected animals (in g/m; non-vac/cs-: 78.1 ± 6.39, vac: 74.6 ± 9.39, non-vac/cs+: 71.6 ± 6.98). There were also no significant differences between the mass of the small intestine and the mass of the stomach. Numerically, the mass of the small intestine was lowest in relation to the mass of the stomach in the clinically healthy animals (non-vac /cs-:4.72 ± 0.61; vac: 5.03 ± 0.55; non-vac/cs: 5.07 ± 0.68).

## Discussion

Performance losses in the course of an *L. intracellularis* infection result from lower average daily gains and a feed conversion ratio which is worse [5–8]. The economic losses due to this infection with *L. intracellularis* in pigs are the main issue for the pig industry [1–4].

### Effects on performance

A high feed intake is the basic prerequisite for exploiting the performance potential of pigs [30, 31]. The first consequence of an infection is the reduced appetite and therefore lower feed intake [32]. In the present study, feed intake in the group non-vac/cs+ was only numerically reduced by 10.2% and in group non-vac/cs- by 6.94% compared to the feed intake of vaccinated animals. In the literature, there is up to now no direct comparison between these three types of groups. In a study with an experimental *L. intracellularis* infection, a reduced feed intake was observed in animals aged 28 to 49 days. When groups with different inoculation doses (counts per pig) were compared (7.2 × 10^7^ to 3.8 × 10^5^ and 7.2 × 10^7^ to 2.2 × 10^6^), the reduction in feed intake was 1.27% and 7.85%, respectively [33]. In another study the reduction in feed intake was 3.79% in pigs at 38 to 58 days of age after experimental infection (1.26x10^10^*L. intracellularis* organisms in the inoculum) in comparison with animals treated with 50 ppm tylvalosin for 14 further days after experimental infection [34]. For viral intestinal infections (porcine epidemic diarrhoea = PED), there are also significant effects on performance. After a 4-day acclimatisation period, the infection with PED resulted in a reduced feed intake of 11.5% over a period of 21 days [22]. In a recent meta-analysis on the effects of infections on feed intake due to bacterial infections of the digestive system (mainly *E. coli* in piglets) the reduction was 15% [19]. In conclusion, an infection with *L. intracellularis* in the acute phase seems to have a somewhat lower effect on the feed intake than viral or classical bacterial intestinal infection in piglet rearing. In the present study, the effects of an *L. intracellularis* infection on the feed intake were higher in percentage terms than in the literature [8, 34]. This can be interpreted as the effect of strict division of animals into three groups according to clinical appearance and vaccination status. At the same time, it should be borne in mind that the experimental approach here per se may reflect the natural conditions in the field best because naturally - infected, conspicuous animals have been selected. Experiments with an artificial infection are not possible in the classical sense due to the lack of possibility of cultural cultivation. For that reason usually strains of *L. intracellularis* are freshly harvested from McCoy cells [35–37]. On the other hand, digestibility studies in animals with a natural microbiome for an *L. intracellularis* infection may simulate the conditions in the field better. This is because interactions between the natural composition of the microbiome in pigs and nutrient digestibility are proven [38]. This is the reason why in the present study unlike in conventional studies with experimental infection, animals from a farm with an early field infection were used.

### Growth factor and inflammation

The Insulin-like growth factor I is an important anabolic hormone that mediates growth and development of individuals [39]. IGF-1 is probably also a useful biomarker of growth performance for comparing pigs from the same herd [40]. In the present study no significant differences in the serum levels of IGF-1 were found. In general, it is stated that the variation in hepatic IGF-1 expression is probably due to the natural variability observed in the young growing pigs. Therefore, this might have an influence on the statistically significant gained results [40].

Numerically, the vaccinated animals had the highest mean values (285 ng / mL). Non-vaccinated, clinically healthy animals (− 4.72%) and pigs with clinical signs of infection (− 20.9%) had lower IGF-1 concentrations in the serum at the time of dissection.

From literature it is known that pigs with higher post-weaning average daily gains have significantly increased odds of elevated IGF-1 expression in the liver [39]. For example in the named study, the pig with the highest average daily gain (556 g/day) was 1073 (95% CI: 16.02–71,892) times more likely to have higher IGF-1 expression than the pig with the lowest gain (140 g/day; *P* < 0.01) [39].

In a five-week feeding experiment with piglets on the effects of antibiotics (22.7 ppm of chlortetracycline, 22.7 ppm of sulfamethazine, and 11.4 ppm of penicillin) the levels of IGF-1 in untreated animals were significant lower (− 19.2%; 105 vs. 131 ng/mL *p* < 0.001; [41]). This observation was accompanied by a decrease in average daily gains of 20.6% (*p* < 0.01; [41]). From studies on growing pigs, it has been known for a long time that restrictive feeding is associated with lower IGF-1 levels in the serum [42]. In general, this is in accordance with the results found here with a lower feed intake in clinically conspicuous animals. Therefore, higher IGF-1 concentrations may be the explanation for the numerically higher feed consumption and the increased average daily gains in the vaccinated group.

### Nutrient digestibility and morphometry of organs

One of the characteristics of an infection with the pathogen *L. intracellularis* in pigs is a downregulation of various genes in the enterocytes. These play an important role in the mechanisms of nutrient absorption [21]. Therefore, it can be assumed that an infection with *L. intracellularis* can have an influence on the digestibility of nutrients. No significant differences were found in the study with regard to the precaecal digestibility of the nutrients, although numerically the digestibility rate of nitrogen decreased by 2.3% percentage points in the presence of clinical manifestations of the disease (non-vac/cs + vs. non-vac/cs-). Until now, there is no comparative study dealing with the influence of this relevant infectious agent of the pig on digestibility of nutrients [22]. For porcine reproductive and respiratory syndrome virus, porcine epidemic diarrhoea virus and a co-infection from both illnesses no significant influence on the ileal digestibility was seen [22]. This is consistent with results found here.

The response of the gastrointestinal tract to ingested nutrients is a complex and closely controlled process [24]. A leading mechanism of intestinal adaptation to enteral availability nutrients is driven by humoral factors like the intestinotrophic peptide glucagon-like peptide-2 (GLP-2) [25]. Enteroendocrine L-cells secrete GLP-2 after direct stimulation by nutrients present in the ileal digesta [25]. GLP-2 is responsible for the adjustment control of the intestinal absorption area and nutrient availability [43]. Within a few days massive adjustments are possible [43]. For example, a reduction in the mass of the small intestine in rats within 6 days could be detected in 34% of cases with parenteral nutrition [43]. Therefore, low digestibility rates of nutrients in the small intestine cause the intestine to react with a morphometric adjustment in the form of growth. This could be the explanation for the fact that a disease might not directly influence the precaecal digestibility of nutrients. However, the result of this adaptation is a longer intestine or higher relative intestinal mass or both. Thus, the relative efficiency of the digestive organs per se might be reduced. Indications for such organ adaptations could also be found in this study. Relative to the length of the small intestine, the clinically conspicuous animals had the smallest body mass and relative to the organ mass of the small intestine, they had, numerically, the smallest body mass. In relation to the organ length, the group of clinically conspicuous animals had significantly lower average daily weight gains.

In the present study, it was seen that average total tract digestibility of nitrogen was significantly reduced in clinically affected animals. This was also demonstrated in the above-mentioned study for the combined infection of PRRSV and PED [22]. The total digestibility of the dry matter, nitrogen and the gross energy was also reduced [22]. While in the aforementioned study the reduction in nitrogen digestibility was 12.8% [22], it was only 3.81% for the clinically conspicuous animals in the present study.

It can be presumed that mucosal alterations might lead to increased presence of nitrogen compounds in the intestine as hypothesised for *Brachyspira* infection in swine [44]. This might be due to the increased amounts of endogenous nitrogen in the distal parts of the small intestine and parts of the large intestine. It is known that *L. intracellularis* is also a prevalent cause of naturally acquired colitis in pigs. About 25% of endogenous nitrogen originates from the large intestine while 59.9% originates from the small intestine [45]. The reabsorption of endogenous nitrogen (total 10.7 g) amounted to 79% towards the end of the small intestine and 88% for the whole digestive tract [46] for an nitrogen-intake of 23.6 g/day. Other authors calculated for higher endogenous secretion (18.1 g for 40 g nitrogen-intake a day [47] and 16.1 g for 35 g nitrogen-intake [48] with reabsorption up to the end of the digestive tract from 90% or rather 78%). Therefore a lower reabsorption of endogenous nitrogen could be a reason for a lower digestibility of nitrogen in clinically apparent piglets.

## Conclusion

This study clearly shows that the performance of pigs is adversely affected by an *L. intracellularis* infection. The lower average weight gain is primarily caused by a lower feed intake. The precaecal digestibility of the nutrients was not reduced significantly. Adaptation mechanisms lead to increased length and relative mass of the small intestine in diseased animals. Therefore, the relative efficiency of nutrient utilisation might be adversely affected. In the case of vaccinated animals, the lowest effects of an *L. intracellularis* infection were observed concerning the performance of the animals.

## Abbreviations

ADFI: Average daily feed intake
ADG: Average daily gains
ATTD: Apparent total tract digestibility
DK: Danish Landrace 50% x Yorkshire 50%
DM: Dry matter
FCR: Feed conversion ratio
GLP-2: Glucagon-like peptide-2
IGF-1: Insulin-like growth factor 1
ME: Metabolizable Energy
Non-vac/cs-: Not vaccinated, clinically no signs
Non-vac/cs +: Not vaccinated, clinical signs
PRRSV: Porcine Reproductive and Respiratory Syndrome Virus
PED: Porcine epidemic diarrhoea
ST: *Salmonella typhimurium*
Vac: vaccinated with Enterisol^®^Ileitis
